# AI-assisted structural consensus-proteome prediction of human monkeypox viruses isolated within a year after the 2022 multi-country outbreak

**DOI:** 10.1128/spectrum.02315-23

**Published:** 2023-10-24

**Authors:** Lena Parigger, Andreas Krassnigg, Stefan Grabuschnig, Karl Gruber, Georg Steinkellner, Christian C. Gruber

**Affiliations:** 1 Innophore, Graz, Austria; 2 Institute of Molecular Biosciences, University of Graz, Graz, Austria; 3 Austrian Centre of Industrial Biotechnology, Graz, Austria; 4 Field of Excellence BioHealth, University of Graz, Graz, Austria; 5 Innophore, San Francisco, California, USA; Institute of Molecular Biology, Academia Sinica, Taipei, Taiwan

**Keywords:** monkeypox, MPX, epidemic, viral proteome, viral genome, homology modeling, AlphaFold2, ESMFold, BioNeMo, structure prediction, structural genomics, consensus genome, tecovirimat, brincidofovir

## Abstract

The monkeypox virus (MPX) belongs to the *Orthopoxvirus* genus of the Poxviridae family, is endemic in parts of Africa and causes a disease in humans similar to smallpox. The most recent outbreak of MPX is already affecting 110 countries, with 86,956 confirmed cases since May 2022 and has consequently become a focus of interest. In particular, a molecular understanding of the virus is essential to study infection processes and pathogen-host interactions, predict tropism changes, or guide drug development and drug discovery as well as vaccine development or vaccine adaptation at a very early stage. Herein, we present a study of the structural proteome of the currently circulating MPX: Our consensus analysis of 3,713 genome sequences sampled within a year after the outbreak revealed 10,580 characteristic candidate open reading frames (ORFs). A search in the non-redundant protein database reduced the number of suspected ORFs to 1,079, of which 210 are representative proteins in typical MPX reference genomes. This should serve as a collection of putative proteins within the currently spreading MPX, a compound of information that could support timely drug discovery, mutational analyses, and vaccine development. We, herein, present the so far most comprehensive structural proteome by providing atomistic 3D models of 210 proteins, generated with three state-of-the-art structure prediction methods, including a mutational analysis of the proteome, with a particular focus on the drug-binding sites of tecovirimat and brincidofovir.

**IMPORTANCE:**

The 2022 outbreak of the monkeypox virus already involves, by April 2023, 110 countries with 86,956 confirmed cases and 119 deaths. Understanding an emerging disease on a molecular level is essential to study infection processes and eventually guide drug discovery at an early stage. To support this, we provide the so far most comprehensive structural proteome of the monkeypox virus, which includes 210 structural models, each computed with three state-of-the-art structure prediction methods. Instead of building on a single-genome sequence, we generated our models from a consensus of 3,713 high-quality genome sequences sampled from patients within 1 year of the outbreak. Therefore, we present an average structural proteome of the currently isolated viruses, including mutational analyses with a special focus on drug-binding sites. Continuing dynamic mutation monitoring within the structural proteome presented here is essential to timely predict possible physiological changes in the evolving virus.

## INTRODUCTION

The monkeypox virus (MPX) was initially isolated from orangutans in an Indonesian zoo by Rijk Gispen (1, 2) in 1949. Its description as a member of the pox family of viruses occurred almost 10 years later, in 1958, and was published by Magnus et al. in 1959 (2, 3). The approximately 197-kbp-long genome of MPX shows over 96% identity to the smallpox variola virus (VAR). Despite their strong relationship, neither does MPX seem to be the ancestor of VAR nor vice versa (4). A previous analysis of the genome in 2002 revealed 190 open reading frames (ORFs), which largely correspond to the essential gene set of the *Orthopoxvirus* genus but differ, e.g.*,* in the equipment with immunomodulatory and host range genes (5, 6).

While being discovered in primates, the original reservoir of MPX is considered to be rodents such as different species of squirrels or striped grass mice (7 – 9). Transmission of the virus to human hosts was first described in 1970 in the Democratic Republic of the Congo (4, 9, 10). In humans, it causes a zoonotic disease (11) with clinical manifestations similar to smallpox disease (9). Since the first transmission, repetitive outbreaks in human populations have mainly been reported from within the Congo basin. However, the disease was spread outside the African continent as well, where a general rise of cases is thought to relate to the suspension of the smallpox vaccination program (12, 13).

The recent outbreak of MPX in 2022 already involves, by April 2023, 110 countries with 86,956 confirmed cases and 119 deaths, according to the Centers for Disease Control and Prevention (https://www.cdc.gov/poxvirus/mpox/response/2022/world-map.html), and has been declared a public-health emergency of international concern (PHEIC) by the Word Health Organization Director-General in July 2022 (14). By definition (15), an outbreak is a sudden increase in the incidence of a disease where the number of cases exceeds the normal expectation for the geographic location and season. Current treatment options against MPX include tecovirimat and brincidofovir, which were earlier approved by the Food and Drug Administration for the treatment of smallpox (16).

In response to such outbreaks, prompt reactions are essential so that therapeutic and non-therapeutic countermeasures can be taken early, even before a PHEIC, epidemic, or pandemic status is declared. Structural information on the viral proteome supports studies of molecular mechanisms related to disease, drug repurposing and drug discovery, as well as vaccine development or vaccine adaptation. This was our main motivation for taking fast action and publishing the structural proteome of the first available MPX genome sequence draft as a preprint (17) within 6 days after the sequence was published on 20 May 2022 (18). In the following months, research groups published proteome predictions via AlphaFold2, including 178, 190, 190 (19), and 186 (20) protein sequences retrieved from single-genome sequence information deposited in the National Center for Biotechnology Information (21) (NCBI) (NC_003310.1, 1996; MT903345.1, 2018; and ON563414.3, 2022) and Uniprot (22) (UP000101269, 1996), respectively. However, due to variations and mutations, studies based on a single-genome sequence might not represent the majority of virus variants currently circulating.

Furthermore, ambiguous bases (usually denoted as “*N*” in the sequence) and sequencing gaps will, in general, decrease the quality of predicted protein sequences. For example, we found that 8.01% of the first genome sequence draft contained ambiguous bases. As a consequence, proteins subjected to homology modeling in the preprint we published contained a number of unknown amino acids (“X”) up to 660 (median: 1 + 8.5 − 1). Note: Since most distributions encountered in our analysis are highly skewed, averages are always given as the median plus the distance to the upper quartile minus the distance to the lower quartile, here and in the entire manuscript.

Due to immense sequencing efforts, more than 7,000 MPX genome sequences were deposited in the publicly accessible database "Global Initiative on Sharing All Influenza Data" (GISAID) (23) by April 2023. This enabled us to perform an extensive mutational analysis of the emerging MPX and to predict the structural consensus proteome, which should accurately reflect the state within 1 year after the 2022 outbreak. To the best of our knowledge, we present the most comprehensive structural proteome of the currently spreading MPX to date by providing 3D structures of 210 MPX reference proteins generated with the AI-guided *de novo* prediction tools AlphaFold2 (24) from DeepMind and ESMFold (25) from Meta, in parts employing NVIDIA’s BioNeMo structural modeling platform, as well as Innophore’s comparative modeling pipeline, whenever applicable. Providing not only AlphaFold2 models (as it is the case in prior studies mentioned above) but also ESMFold and homology models allows to explore possible structural deviations derived from different modeling algorithms. Notably, homology models may represent oligomeric states, as well as include cofactors, ligands, or DNA/RNA molecules. These not only give insights into potential binding properties or functions but also certainly influence the structural composition of the protein, such as structural changes upon ligand or DNA binding, or the formation of protein pores through oligomerization. Furthermore, with the intention to produce structural models which represent the functional state of proteins, we provide the structure of protein sequences without potential signal sequences, while the previous studies provide structural models of complete ORFs.

In addition to structure prediction, we performed a mutational analysis of the proteins by comparing 7,023 publicly available genome sequences (23) sampled from April 2022 to April 2023. We are confident that our data will support the scientific community’s and pharmaceutical industry’s responses to the putative risks of the MPX outbreak.

## RESULTS

### The consensus genome sequence

To overcome the disadvantages of sequencing errors and ambiguous bases (denoted as “*N*”) and, furthermore, to best represent the current state of the outbreak, a consensus genome was built from a curated set of 3,713 MPX genome sequences available as a multiple-sequence alignment (MSA) at GISAID (23), isolated from human samples between April 2022 and April 2023. These sequences contain 0.00%–3.98% ambiguous bases (median: 0.02 + 0.55 – 0.02%) and have lengths of 147,759 – 197,205 bps (median: 197,110 + 48 – 509 bps). The resulting consensus genome sequence contains 197,164 bps without any ambiguous bases, and its conservation ranges from 55.62% to 100% (median: 99.87 + 0.08 – 0.27%).

Notably, a consensus sequence is heavily dependent on the underlying genome sequences. Among the 3,713 sequences we have considered, the majority, totaling 3,154 isolates, were collected from June 2022 to September 2022 (inclusive). Consequently, the consensus is largely influenced by the viruses circulating at that time. In contrast, little sequence data were available at the very beginning of the outbreak (231 in April and May 2022) and after September 2022 (328 isolates). A consensus sequence built from limited data is more likely to be influenced by sequencing errors or mutations in genomes, which might occur quite rarely in the viral population and do not present the actual current evolutionary state of the virus. This rationale further led to our decision to incorporate all sequences within the MSA that were sampled throughout the 2022 outbreak, which enhances the robustness of our consensus analysis.

The consensus sequence and the proportion of characters (“A,” “G,” “T,” “C,” gaps, or “N”) at each position are available in the Supplementary Information (see Data S1 and S2 at https://doi.org/10.6084/m9.figshare.22730459.v5).

### Identification of putative ORFs in the consensus genome

In an attempt to obtain a comprehensive view of all theoretically possible protein sequences contained within the consensus genome, the sequence was scanned for ORFs with a minimum length of 10 amino acids, covering the forward and reverse strand in all three reading frames. To filter the resulting 10,580 distinctive ORFs for potential protein sequences, they were used as query sequences for a search in the non-redundant protein (nr) database (26) using the Basic Local Alignment Search Tool (27, 28) (BLASTP), as well as a set of reference proteins derived from the proteomes of NCBI reference genomes of MPX, NC_063383.1 and MN648051.1 (the reference proteome, Data S3, is available at https://doi.org/10.6084/m9.figshare.22730459.v5). An additional BLASTP search in the Protein Data Bank (29) (PDB) was performed in order to identify suitable sequences for homology modeling.

These searches resulted in 1,079 unique ORFs matching to the nr database, 169 matching to the PDB, and 210 matching to the NCBI reference proteome (Table 1). The theoretical consensus ORFs, as well as their matching results to the nr database, the PDB, and the NCBI reference proteome, are included in the Supplementary Information (see Data S4 at https://doi.org/10.6084/m9.figshare.22730459.v5).

**TABLE 1:** BLASTP results for the search of 10,580 distinctive ORFs in different databases^*a*^

Search database	Number of unique ORFs matching	Sequence identity (%)	ORF coverage (%)
nr	1,079	21.25 | 44.19 | 80.00 | 98.89 | 100	30.51 | 74.46 | 88.89 | 97.89 | 119.51
NCBI ref	210	97.67 | 100 | 100 | 100 | 100	52.45 | 94.13 | 98.55 | 100 | 100
PDB	169	19.81 | 30.85 | 51.85 | 95.00 | 100	9.62 | 43.17 | 76.82 | 95.28 | 120.63

^*a*^ Sequence identity and coverage of ORFs in the alignment are given in quartiles (minimum | lower quartile | median | upper quartile | maximum).

### Length- and conservation analysis of theoretical ORFs

Matching ORFs to databases, as described above, is one way of distinguishing functionally expressed proteins (which match to proteins of the organism’s reference proteome) or evolutionary relics (matching to existing databases of protein sequences, like the nr database) from parts of the genome, which are neither expressed nor evolutionary relevant and thus not present in protein databases. Nevertheless, reference proteomes could be incomplete, potentially leading to the presence of functionally expressed proteins extending beyond those ORFs matching the reference proteome. Moreover, the collection of 10,580 theoretical ORFs present within the consensus genome might contain yet undiscovered protein sequences. These sequences cannot be detected by a BLAST search against existing databases. In order to identify such sequences, we conducted a length- and conservation analysis of all ORFs, with the aim of finding patterns to distinguish potentially functional or evolutionary relevant ORFs from the rest.

**Fig 1:**
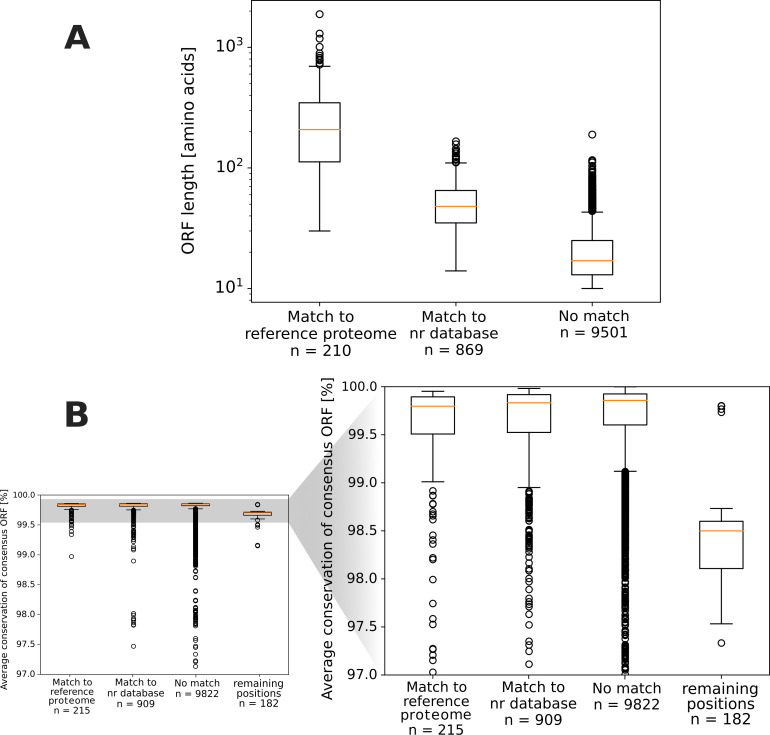
Length distribution (**A**) and average conservation (**B**) of putative ORFs in the consensus genome sequence. ORF lengths (in amino acids) are displayed in (A) for ORFs matching to the NCBI reference proteome, the nr database (excluding ORFs matching to the reference proteome), and not matching any proteins in these databases. The average conservation (in %) across the ORFs (including stop codons) is displayed in (B), along with the conservation of 182 nucleotide bases not included in any of the theoretical ORFs. Notably, the number of ORFs (*N*) in (A) is lower than in (B) because (A) includes only distinct ORFs and (B) treats all identified ORFs (also duplicated regions) as single elements.

The ORF-length distribution shows significant differences between ORFs matching to the NCBI reference proteome, the nr database, or not matching at all (Fig. 1A). ORFs matching to existing databases (like the nr database) are likely remnants of viral evolution. Hence, it was expected that they would exhibit longer sequence lengths compared to portions of the translated genome that do not match to sequences within databases and thus most probably represent non-functional regions in the genome, which is shown in Fig. 1A. Such non-functional regions are expected to have closely spaced stop codons, hence, would translate into short amino-acid sequences, to prevent the generation of extensive non-sense proteins in case there is a mistake during the translation process which leads to an unintentional frameshift. Based on these observations, one could already, by their sequence length, make assumptions about which of the theoretical ORFs are more likely to correspond to functionality, assuming that the reference proteome consists of proteins, which are actually functionally expressed during the viral infection cycle.

We were particularly interested in the outlier in Fig. 1A’s rightmost boxplot, ID_5764 with 189 amino acids because of its notably extended sequence length for a sequence not matching sequences in existing databases. This led us to consider the possibility that it might be a yet unrecognized protein sequence, either as an evolutionary relic or as a potentially expressed protein. Although it holds true that other non-matching ORFs could also be traces of evolution or potentially functional protein sequences, we assume that the likelihood of this increases with the ORF’s length. Consequently, our focus centers on ID_5764. In an attempt to find homologs, we conducted an additional BLASTP search of ORF ID_5764 in the comprehensive MGnify database (30), which contains more than 2.4 billion non-redundant sequences. However, this also resulted in no homologous matches to this sequence. We provide its 3D structure, predicted with AlphaFold2 (24) and ESMFold (25), as well as potential binding sites calculated with the Catalophore platform (31) in the Supplementary Information (Fig. S1, structural models available at https://doi.org/10.6084/m9.figshare.22730459.v5). Note that, due to the lack of homologous sequences to ID_5764, the two structure prediction tools resulted in low-confidence structures with substantial differences in their overall fold.

Despite our initial assumption that the sequences of functionally expressed proteins would generally have higher conservation than ORFs which are not functionally expressed, no significant difference was noticed in the average conservation across ORF sequences matching to reference proteins, the nr database, or not matching at all (Fig. 1B). This might be explained by the fact that ORFs were collected from all six reading frames. Therefore, mutations identified in ORFs matching to databases were also identified in non-matching ORFs, if present at the same position but in other reading frames, and vice versa. If, as we expected, functionally expressed proteins are generally more sensitive to mutations than other ORFs, this would not directly display a different level of conservation between these sequences, as the sensitivity would also influence the presence of this mutation in ORFs in the other reading frames at this position. Notably, genome positions, which are not included in the total of theoretical ORFs in all six reading frames (referred to as “remaining bases”), mostly show lower conservation than sequences identified as ORFs.

### 3D structure prediction of the consensus proteome

In the context described above, we predicted the structures of those consensus ORFs that matched with the NCBI reference proteome, as this similarity implies a higher likelihood of functional expression by the virus, in order to provide 3D information on the proteins that represent the currently circulating MPX as faithfully as possible. Within this selection of 210 protein sequences, 26 sequences (ID_9820, ID_9811, ID_8973, ID_8682, ID_7894, ID_7637, ID_7168, ID_7151, ID_6070, ID_6_and_ID_5474, ID_5944, ID_5868, ID_5387, ID_5347, ID_5315, ID_5201, ID_5180, ID_5163, ID_5080, ID_3541, ID_3347, ID_2009, ID_1649, ID_1498, ID_1156, and ID_10869) were newly predicted as opposed to the previous studies providing AlphaFold2 models of MPX proteomes (19, 20). Furthermore, in contrast to these prior studies, modeling was performed by AI-guided structure prediction tools AlphaFold2 (24) and ESMFold (25), as well as if an ORF also matched with a protein in the PDB, by homology modeling using the Catalophore DrugSolver Platform employing Yasara (32). Lacking experimentally determined structures of specific proteins (or their variants), we found it advisable to use different 3D models as a template for, e.g., *in silico* drug screening. The reason for this procedure is that we noticed significant variation in the 3D composition of models generated with ESMFold, AlphaFold2, and homology modeling in some cases, with a definite potential to influence the outcome of virtual docking, molecular dynamics simulations, and generative AI pipelines. Notably, homology models may represent oligomeric states, as well as include cofactors, ligands, or DNA/RNA molecules. These not only give insights into potential binding properties or functions but also certainly influence the structural composition of the protein, such as structural changes upon ligand or DNA binding, or the formation of protein pores through oligomerization. In our data set, 10 homology models contain nucleic acid molecules, 61 contain ligands, and 31 homology models predict a multimeric state. Such considerations are absent from the previous studies, which solely provide AlphaFold2 models.

Note that some homology models may only represent fractions of the protein sequence, in contrast to AlphaFold2 or ESMFold models. The exact sequence which is contained in the respective homology model, together with information about the presence of ligands or nucleic acid, and potential multimeric states, is available in the Supplementary Information (see Data S4 at https://doi.org/10.6084/m9.figshare.22730459.v5). We strongly advise taking this information seriously when choosing a specific model for any kind of analysis in order to avoid artifacts resulting from structures of truncated sequences. For protein models generated with AlphaFold2 and ESMFold, the predicted local distance difference test (pLDDT) score, a per-residue confidence score, is represented as the B-factor column in the respective PDB files. The pLDDT value gives relevant information on the reliability of the protein models or different regions within them. Note that values greater than 90 indicate high structure prediction confidence.

**Fig 2:**
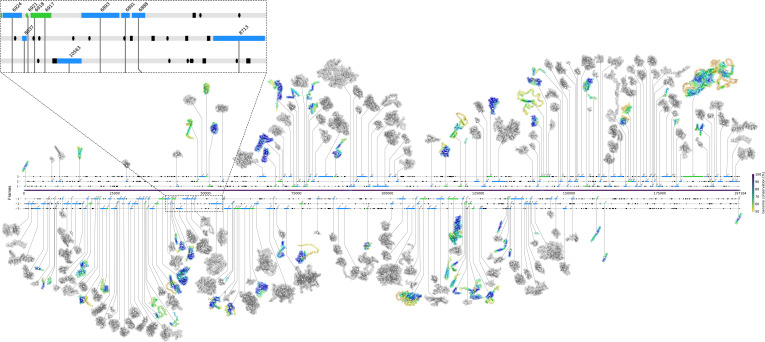
Genomic map of the putative structural consensus proteome. The consensus genome sequence, colored by conservation, is shown (at *y* = 0) along with the identified ORFs in the respective frame (shown on the *y*-axis), which matched to the nr database (black, green, and blue horizontal bars). ORFs that were identified in the forward strand (frames 1 to 3) are depicted above the genome sequence, whereas ORFs identified in the reverse strand (frames −1 to −3) are depicted below. Blue- and green-colored bars represent ORFs which additionally match the NCBI reference proteome. Green-colored ORFs do not match to proteins deposited in the PDB, thus ESMFold and AlphaFold2 models were built. The latter are depicted in the figure, colored by their pLDDT with the PyMOL color range “rainbow_rev,” given a minimum of 0 (red) and a maximum of 100 (dark blue). Blue-colored ORFs do match with proteins in the PDB, and therefore, additionally to AlphaFold2 and ESMFold models, homology models (depicted in gray) were generated.

The results of the structure prediction are shown in Fig. 2, whereas a more comprehensive data set consisting of information on the structural modeling, the distribution of pLDDT scores, matches to databases, and properties (including positions in the genome, topology predictions, and mutational analyses) of all potential ORFs within the MPX consensus genome is available in the Supplementary Information (see Data S4 at https://doi.org/10.6084/m9.figshare.22730459.v5). The protein models generated within this study are available at https://doi.org/10.6084/m9.figshare.22730459.v5.

### Mutational analysis of the consensus proteome

Within the consensus proteome, 26 ORFs contained mutations when compared to the NCBI reference proteome (1–3 amino-acid exchanges per ORF). All of these consensus mutations were also detected in some genome sequences sampled before the 2022 outbreak. More precisely, these mutations appear in 4.23 + 11.01 – 0.05% (range: 3.33%–58.33%) of all respective protein sequences within genomes sampled before and 96.36 + 1.03 – 3.23% (range: 79.38%–98.79%) of all protein sequences detected within 1 year after the 2022 outbreak. Reference proteins in which consensus mutations were detected include the drug targets of currently available antivirals tecovirimat and brincidofovir, namely phospholipase F13 (33, 34) (consensus mutation E353K), also known as VP37, and DNA polymerase E9 (35, 36) (consensus mutation L108F), respectively. Other consensus mutations were detected in proteins involved in immunological mechanisms and replication of the virus.

Besides mutations in the consensus protein sequences, we detected 1–171 (median: 15 + 10 – 6) distinct mutations or mutation combinations within all 7,023 viral isolates for the 210 ORFs which were considered. This means that, besides the consensus mutation, we find at least one other mutation (e.g., for ID_7637: T45C) and at most 171 different mutations or different mutation combinations (e.g., for ID_5375, where the most mutated protein variant contains 35 mutations) within the viral isolates. Consequently, this analysis yields a count of 1–1,379 viral isolates (median: 67.5 + 60.5 – 30.5) neither bearing the consensus mutation nor representing the NCBI reference protein sequence. The number of distinct ORF variants across the viral isolates hereby strongly correlates with the respective ORF’s length. As mutations are in general introduced randomly across the genome, longer ORFs inherently have a greater chance to incorporate mutations than shorter ones (Fig. S2). More details about these results are available in the Supplementary Information (see Data S4 at https://doi.org/10.6084/m9.figshare.22730459.v5).

**Fig 3:**
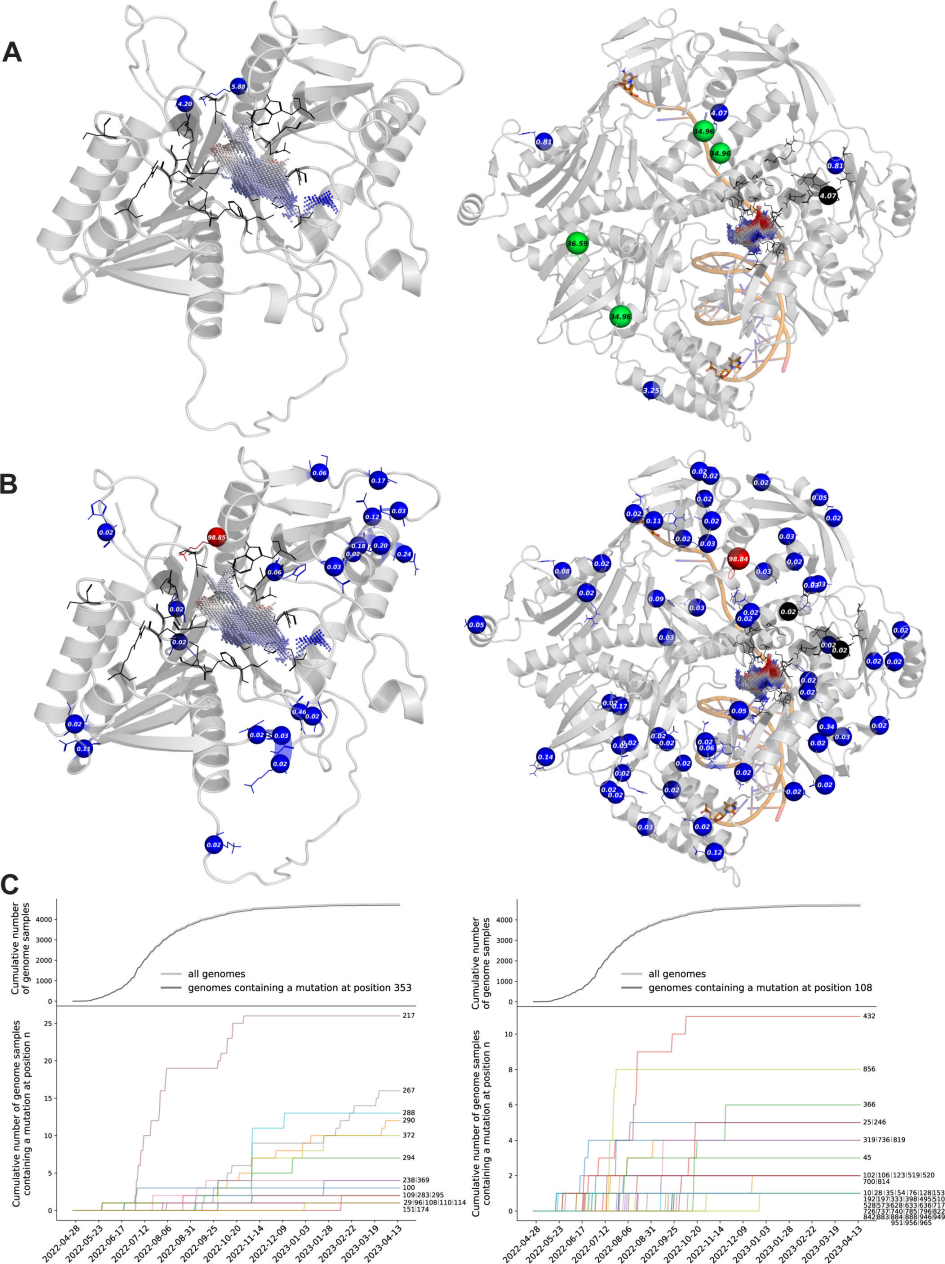
Mutation events in drug targets phospholipase F13 and DNA polymerase. (**A**) Homology model of ORF ID_6924, representing tecovirimat-targeted phospholipase F13 (left) and experimentally determined 3D structure of brincidofovir-targeted DNA polymerase [PDB 8HG1 (39), chains B and C are not shown], referring to ORF ID_8713 (right). Cα-atoms of mutated positions detected before the 2022 MPX outbreak are shown as spheres, colored with the PyMOL “rainbow” palette (minimum = 0, maximum = 100) by the mutations’ frequency (labeled in %) in all genome samples where the respective protein sequence was identified. Residues previously predicted to interact with tecovirimat (37) (left) and brincidofovir (38) (right) are shown as black lines, and the respective binding site cavity is colored by the electrostatics of its surroundings (blue-white-red spectrum ranging from −1 to +1). (**B**) Structural representation as in (A), showing mutated positions detected within 1 year after the 2022 MPX outbreak. (**C**) A cumulative number of genomes detected per day from April 2022 to April 2023, which contain mutations at respective positions (positions labeled on the right of each line) in ID_6924 (left) and ID_8713 (right). The graphs are aligned with the number of genomes containing a mutation at the position of the consensus mutation (top: dark gray) as well as the total number of genomes sampled during the time period (top: light gray). More details on mutation numbers are available in the Supplementary Information (see Data S4 at https://doi.org/10.6084/m9.figshare.22730459.v5).

We show a structural representation of the mutational events in drug targets phospholipase F13 and DNA polymerase E9, referring to query sequences ID_6924 and ID_8713, respectively, with a special focus on predicted drug-binding sites (Fig. 3A and B). A significantly higher number of distinctive mutated positions were detected in genomes sampled during the outbreak (ID_6924: 20 mutations; ID_8713: 59 mutations) compared to before (ID_6924: 2 mutations; ID_8713: 9 mutations), which is most probably a result of the higher number of genomes sampled during the outbreak (6,570 samples) than before (198 samples). None of these genomes showed mutations in ID_6924 at residues that were previously identified to directly interact with tecovirimat (37). On the contrary, mutations at two brincidofovir-interacting positions (38) within ID_8713 were detected, namely R628K (occurring in one genome sequence sampled in July 2022) and N785D (occurring in six genome sequences, with one sampled in May 2022 and five sampled from 2006 to 2007). A time-resolved analysis of mutations in the drug targets ID_6924 and ID_8713 showed that the majority of mutation events occurred from June 2022 to October 2022, which are certainly coupled with the increased number of total genomes sampled during that time and thus underline the importance of extensive genome sequencing for the identification of potentially risk-associated mutations in human pathogens like MPX (Fig. 3C).

To further investigate the mutations’ effects on tecovirimat- and brincidofovir-binding sites, we employed a comparison of binding site properties and shapes among different protein variants using the Catalophore (31) cavity matching technique (Fig. S3 to S6). This approach provides us with insights into how mutations might influence the drug-binding site, even when the exact binding mode of the drug is unknown.

At the time this manuscript was submitted, experimentally determined complex structures of protein-ligand complexes were unavailable for the specific protein-drug combinations which are proposed for the treatment of monkeypox. We, therefore, considered the drug-interacting residues presented in previous studies for tecovirimat (37) and brincidofovir (38) and calculated point clouds representing the cavities at the respective proposed binding sites. We like to emphasize that the Catalophore cavity matching approach has been validated and published, demonstrating that similar binding site cavities in two distinct proteins correspond to their ability to bind the same molecule (40).

We computed homology models of the unique protein sequences parsed from 7,023 genome samples available by April 2023 at GISAID (23) and compared their binding site cavities with the Catalophore (31) technology. While observed differences in cavities might not directly correlate with differences in drug binding, they offer valuable insights into how mutations influence the physicochemical properties of potential drug-binding sites.

Matching of all protein variants of ID_6924 and ID_8713 to their wild-type (i.e., their matching partner in the NCBI reference proteome), as well as their consensus variant (i.e.*,* containing mutations E353K and L108, respectively), resulted in little overall differences in the binding site cavities (Supplementary Information, Fig. S3 and S4), with electrostatics contributing most to these differences (Fig. S5).

In the binding site cavity of ID_6924, the most significant difference was observed when compared to a protein variant containing the amino-acid exchange S207F and lacking the consensus mutation E353K. There is only one genome sequence containing this mutation, which was assigned to an incomplete collection date (2022-00-00). Most certainly, the difference in electrostatics results from the exchange of negatively charged glutamic acid (E) to positively charged lysine (K). Changes in hydrophobicity, however, mostly result from energy minimization of side-chain residues (especially leucine at position 118) during the homology modeling process rather than the effects of the specific amino-acid exchanges in the protein (Fig. S6).

The binding site of consensus protein ID_8713 showed the most difference to protein variant E633K, which also contains the consensus mutation L108F (Fig. S3 and S4). The change in electrostatics is explainable by the exchange of negatively charged E to positively charged K, thus resulting in a shift to positive charges in the binding site (Fig. S5). The mutation E633K occurs only in one viral isolate, which was collected in August 2022.

It is important to clarify that we do not label any of the above mutations as “resistance mutations'.” Instead, our analysis focuses on evaluating the potential impact of the entirety of mutations identified across all viral isolates on drug-binding sites. Eventually, continuing dynamic monitoring of the structural proteome presented here and the potential increase in certain mutations should be considered, especially for drug targets such as phospholipase F13 (33, 34) and DNA polymerase E9 (35, 36). This gets emphasized by a recent study, where we show that mutational dynamics at drug-binding sites may drastically change upon increased deployment of certain therapeutics (41).

## DISCUSSION

Understanding emerging pathogens such as MPX or severe acute respiratory syndrome coronavirus 2 (SARS-CoV-2) on a molecular level is essential to study infection processes and pathogen-host interactions, predict tropism changes, or guide drug repurposing and drug discovery as well as vaccine development or adaptation at a very early stage. While traditional structural biology methods such as NMR, cryo-EM, or X-ray crystallography have advanced significantly in recent decades, it still took a while, despite increased efforts in the midst of a rapid international response to the SARS-CoV-2 outbreak in Wuhan/China in January 2020, until the first experimentally determined (complex-) structures of the SARS-CoV-2 main protease (M^pro^) were published by Yang et al. (42) and later Hilgenfeld et al. (43).

To overcome the limitations of time- and human-resources-consuming experimental methods, structural bioinformatics can provide quick and early insight into the genome from a 3D molecular perspective by predicting genome-wide protein structures for a complete pathogen as soon as its sequence is available. For example, we published the first structural model of SARS-CoV-2 M^pro^ on 23 January 2020 (44), 1 week after the 2019-ncov draft genome was published. This model was in good accordance with the crystal structure (PDB 6LU7), released afterward, featuring a root mean square deviation of 0.6 Å for 282 out of 306 superimposed Cα-atoms.

Interestingly, the binding site identified in that model was finally the drug target site of the majority of today’s approved or investigated SARS-CoV-2 direct-acting antivirals such as Paxlovid and others (45, 46). On a larger scale, early predictive structural genomes enable large-scale virtual screening for drug repurposing or development of new drugs (47, 48), a deeper understanding of viral evolution and its structural implications, and prediction of the significance and impact of emerging viral variants (49, 50).

In May 2022, we took fast action in predicting the structural proteome of MPX and publicly provided 3D structural models only 6 days after the first genome draft (18) of the emerging pathogen was communicated. Ambiguous bases and sequencing gaps, which are a common result of sequencing, resulted in lower quality of predicted ORFs. Furthermore, mutations within this first genome draft might not be a representative of the actual or current virus population. Thanks to immense sequencing efforts and publicly available databases, we are now able to provide a set of theoretical ORFs contained in the consensus sequence of 3,713 high-quality MPX genomes, including 210 structural models created with up to three state-of-the-art prediction tools. At the same time, we can point out mutational variations within 7,023 genome samples available in GISAID (23) by April 2023 and present possible mutational effects on drug-binding sites in phospholipase F13 (33, 34) and DNA polymerase E9 (35, 36).

Continuing surveillance of mutational dynamics will enable vaccine- and drug developers to monitor and adapt their candidates to emerging variants, if required (51). Overall, structural-bioinformatics pipelines (52), in combination with transparently shared open scientific data, have proven to be an essential early-response tool for outbreaks (53). The set of 210 structures generated from high-quality and representative protein sequences presented in this work should serve as a collection of putative proteins within the currently spreading MPX, a compound of information that could support timely drug discovery, mutational analyses, and vaccine development. The remaining theoretical ORFs, which did not match the reference proteome, may contain fractions of protein sequences involved in the evolutionary origin of this virus. Besides that, they may include as-yet-unidentified physiological proteins of MPX. In the context of pandemic preparedness, the compilation of such potential protein sequences is useful as it facilitates the identification of proteins that could unexpectedly be expressed by the virus under specific conditions or if the virus evolves to express novel proteins. We, herein, present the complete potential inherent in the monkeypox genome. It is to note that the task of confirming which of the predicted ORFs are functionally expressed lies beyond the scope of this particular study.

## MATERIALS AND METHODS

### Sequence data

The analyses presented herein are based on publicly available genome sequences downloaded from GISAID (23) on 25 April 2023. The data set includes 7,023 genomes with lengths ranging from 298 to 228,869 bps (median: 196,453 + 712 – 805 bps), with 0.00%–99.05% ambiguous bases (median: 0.31 + 1.22 – 0.30), denoted as “*N.*” For the generation of the consensus genome sequence and resulting theoretical ORFs, an MSA file provided by GISAID was used, including 3,713 genome sequences. This file is already cured from low-quality sequences to allow for a meaningful alignment, which shows in the distribution of sequence length and ambiguous bases when comparing to all 7,023 viral isolates. The MSA includes sequences of length 147,759–197,205 bps (median: 197,110 + 48 – 509 bps) with 0.00%–3.98% ambiguous bases (median 0.02 + 0.55 – 0.02%).

Within all 7,023 viral isolates, 198 genomes were sampled before the 2022 outbreak (1962-00-00 to 2022-03-00), and 6,570 were sampled during the outbreak (starting with 2022-04-28). For time-dependent analyses, 255 genome sequences were excluded because they could not be assigned to a time before or after the start of the outbreak due to incomplete dates (2022-00-00). An acknowledgment table, including accession numbers and the origin of the processed sequences, is included in the Supplementary Information (see Data S5 at https://doi.org/10.6084/m9.figshare.22730459.v5).

### Generation of the consensus genome sequence

In order to best reflect the majority of virus variants currently circulating since the 2022 outbreak, a consensus genome was built from 3,713 high-quality genomes provided in an MSA at GISAID, which were sampled within the first year of the 2022 outbreak. In-house Python code, employing the Biopython 1.79 (54) package Bio.SeqIO, was used to build the consensus by determining the most abundant character, including gaps and ambiguous bases (“A,” “T,” “G,” “C,” “N,” or “-”) at each position in the MSA. The resulting sequence contains 197,164 bps without any ambiguous bases, and its conservation ranges from 55.62% to 100% (median: 99.87 + 0.08 – 0.27%). The consensus genome sequence and the distribution of all characters at each position are available in the Supplementary Information (see Data S1 and S2 at https://doi.org/10.6084/m9.figshare.22730459.v5).

### Identification of potential ORFs in MPX genome sequences

As bioinformatic molecular interaction studies rely on protein models, we developed a pipeline embedded in the Catalophore DrugSolver platform, employing the Biopython (54) package Bio.SeqIO, to quickly process genome sequences and provide a set of potential proteins, a putative proteome.

The first step consists of translating both the reverse and forward strand in three reading frames in order to account for every possible translation frame. Therefore, the set of ORFs considered initially also includes cases of overlapping genes, shifted translation starts, and AUG-codon-independent translation initiation (55). Subsequently, the translated sequences are split at stop codons (TAA, TAG, TGA), and only ORFs consisting of a minimum of 10 amino acids are accepted.

In this study, 10,946 theoretical ORFs, of which 366 are duplicates, were identified within the consensus genome. The ORF scan of all 7,023 genomes, which were available by April 2023, resulted in a range of 19–21,904 ORFs (median: 10,819 + 127 – 198 ORFs), containing in total 0%–99.19% (median: 0.40 + 1.54 – 0.38%) unknown amino acids (“X”), which result from the translation of codons containing ambiguous bases.

The number of potential consensus ORFs was further limited to protein sequences with an enhanced possibility to be part of the physiological MPX proteome by performing command-line-based BLASTP (27, 28) searches with default options in the nr database (26), resulting in 1,079 distinct matches. An additional search in the PDB identified 169 ORFs that qualified for structure prediction via comparative modeling.

Since MPX proteomes are available in public databases such as NCBI (21), we further prepared a set of consensus reference proteins, from which sequences were used for modeling the consensus structural proteome. The reference proteome was additionally used to perform a comprehensive time-dependent mutational analysis of the reference proteins within the available MPX genomes, differentiating between those sampled before and within the 2022 outbreak. In this process, the ORFs resulting from the initial ORF scan of each genome were compared to a reference proteome, which consists of 179 proteins contained in the NCBI Reference Sequence NC_063383.1 (collected in Nigeria, 2018), extended with additional 32 proteins contained in MN648051.1, a reference genome sampled during the MPX outbreak in Israel (2018). For reference, this NCBI proteome set is available in the Supplementary Information (see Data S3 at https://doi.org/10.6084/m9.figshare.22730459.v5).

### Modeling of the consensus reference proteome

The BLASTP search of 10,580 theoretical consensus ORFs in the reference proteome set (including proteins from NCBI reference genomes NC_063383.1 and MN648051.1) resulted in 210 matches with a sequence identity ranging from 97.67% to 100% (median: 100 + 0.00 – 0.00%) and a query coverage ranging from 52.45% to 100% (median: 98.55 + 1.45 – 4.42%). For structural modeling, the part of the ORF sequences which did not align to the reference proteins was omitted to prevent modeling artificial *N*-terminally prolonged sequences. Such *N*-terminally prolonged sequences will naturally result from the ORF identification, except if the first codon upstream of the ORF is a stop codon. Prior to structure prediction, the command-line-based deep-learning protein language model DeepTMHMM (56) was applied for predicting topology and signal sequences in the consensus reference ORFs. Topology predictions for each of the 210 proteins are available in the Supplementary Information (see Data S4 at https://doi.org/10.6084/m9.figshare.22730459.v5).

We modeled the structure of 210 consensus reference proteins, excluding predicted signal sequences, using AI-guided structure prediction tools AlphaFold2 (24) and ESMFold (25), in parts employing NVIDIA’s BioNeMo, as well as, for 148 ORFs which matched to sequences deposited in the PDB with sufficient sequence alignment, by homology modeling using the Catalophore DrugSolver Platform employing Yasara (32). Homology modeling was performed with 6 PSI-BLAST iterations, a maximal expect value of 0.5, 5 templates to consider, a maximum of 5 alignment variations per template, and 50 conformations tried per loop. We did not model terminal ORF residues that protrude beyond the alignment with the matched protein in the PDB. AlphaFold2 and ESMFold predictions were performed with ColabFold using the default settings (57). For each protein sequence, five AlphaFold2 models were generated. Out of these five, the model with the highest pLDDT, along with ESMFold and homology models, is deposited at https://doi.org/10.6084/m9.figshare.22730459.v5.

### Mutational analysis of protein sequences

In order to detect any mutations compared to the NCBI reference proteome within 7,023 MPX genome sequences, each ORF’s amino-acid sequence was searched in the reference proteome via BLASTP using the Biopython 1.79 (54) package’s Bio.pairwise2 module. To ensure the correct mapping of a mutation to its position in the protein sequence, only high-quality amino-acid exchanges were included for the subsequent analysis. These refer to exchanges not directly adjacent to deletions, insertions, or uncertain residues (indicated with “X”) and with a surrounding of 10 residues on each side of the mutation, which, if this region contains a deletion or insertion, includes at most 50% mismatches of any kind.

The mutations’ effects on drug-binding sites were evaluated by comparing 3D point clouds representing binding site cavities in homology models of the different protein variants using the Catalophore platform (31). Point clouds were created employing the LIGSITE algorithm (58) with a cutoff value of 5. Details on point cloud matching are mentioned in our previous publication (50) (Materials and Methods section “Catalophore Halo analysis”).

Time-resolved analysis of mutation events was performed by assigning the collection dates of genome sequences retrieved from GISAID (23) to the respective protein sequences. The data set contained 255 genomes with incomplete collection dates (2022-00-00), which were consequently excluded from the time-dependent analysis.

### Visualizations

Data were plotted using the Matplotlib (59) package in Python. Structural representations were created with Pymol 2.5.2 (Schrodinger Inc., Open Source, https://pymol.org) and the CavitOmiX plugin (Innophore, https://innophore.com/cavitomix). Individual visualizations were combined to composite figures in post-processing.

## Data Availability

Publicly available genome sequences were downloaded from https://www.gisaid.org/. An acknowledgment table, including accession numbers and the origin of the processed sequences, is included in the Supplementary Information (see Data S5 at https://doi.org/10.6084/m9.figshare.22730459.v5). The genome sequences herein used as reference sequences are available at https://www.ncbi.nlm.nih.gov/nuccore/NC_063383.1/ and https://www.ncbi.nlm.nih.gov/nuccore/MN648051.1/. Additional supporting data, as well as the data set containing process parameters and properties of all theoretical consensus ORFs (including mutational analyses) and protein structural models is provided in the Supplementary Information and at https://doi.org/10.6084/m9.figshare.22730459.v5. The code used for the data preparation and analysis described herein is available on GitHub at https://github.com/innophore/virus.watch-mpx. Python scripts for mutational analyses of proteins are available at https://github.com/innophore/virus.watch-mpro.

## SUPPLEMENTAL MATERIAL

Supplemental figures spectrum.02315-23-s0001.pdf Fig. S1 to S6.

ASM does not own the copyrights to Supplemental Material that may be linked to, or accessed through, an article. The authors have granted ASM a non-exclusive, world-wide license to publish the Supplemental Material files. Please contact the corresponding author directly for reuse.
