# Supplementary material for: AI-assisted structural consensus-proteome prediction of human monkeypox viruses isolated within a year after the 2022 multi-country outbreak
Source: Microbiol Spectr. 2023 Oct 24;11(6):e02315-23. doi: 10.1128/spectrum.02315-23 (PMC10714838; doi:10.1128/spectrum.02315-23)
Supplement: Supplemental figures — Fig. S1 to S6. [file spectrum.02315-23-s0001.pdf]

Supplementary Information

AI-assisted structural consensus-proteome prediction of human monkeypox viruses isolated within a year after the 2022 multi-country outbreak

Lena Parigger[1,2], Andreas Krassnigg[1], Stefan Grabuschnig[1], Karl Gruber[1,2,3,4], Georg Steinkellner[2,4,5] & Christian C. Gruber[2,3,4,5*]

[1]Innophore, 8010, Graz, Austria

[2]Institute of Molecular Biosciences, University of Graz, 8010, Graz, Austria

[3]Austrian Centre of Industrial Biotechnology, Graz, Austria

[4]Field of Excellence BioHealth, University of Graz, Graz, Austria

[5]Innophore, San Francisco, CA, USA

*Correspondence should be addressed to (christian.gruber@innophore.com)

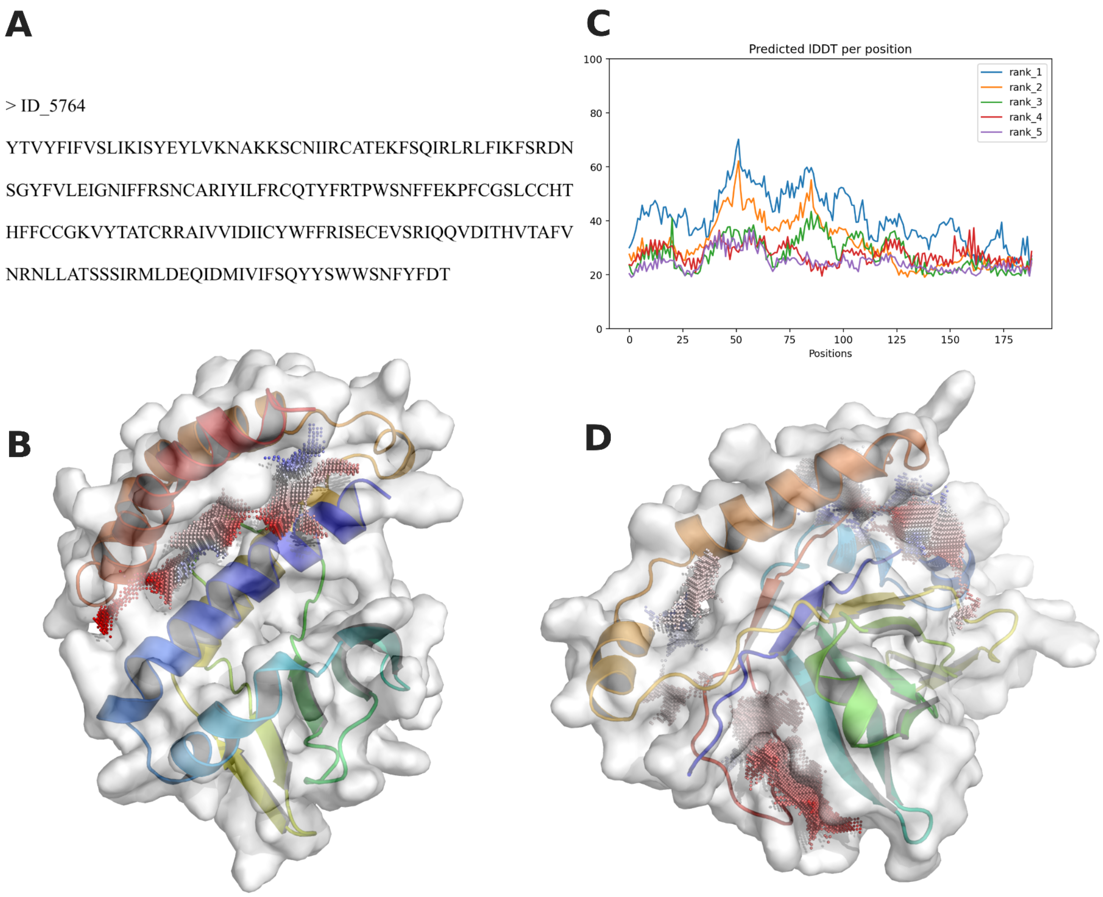

Figure S1 | Structural models of ORF ID_5764 and potential binding sites. A) Sequence of ID_5764 which was subjected to structure prediction. B) Structure prediction of ID_5764 by ESMFold[1], shown as a cartoon colored in the PyMOL "rainbow" spectrum from blue (*N*-terminus) to red (*C*-terminus). The protein surface is shown in gray. A 3D point-cloud representing a potential binding pocket was calculated using the Catalophore™ platform[2] and is colored by the electrostatics of its surroundings (blue-white-red spectrum ranging from -1 to +1). C) Confidence values (plDDT) of the AlphaFold2[3] model along the sequence of ID_5764. Five models were built and ranked by their plDDT (rank 1-5), of which the one with the highest overall plDDT is depicted in D). D) Structure prediction using AlphaFold2, structural representation as in B). Three potential binding sites were detected, represented by 3D point-clouds colored by electrostatics.

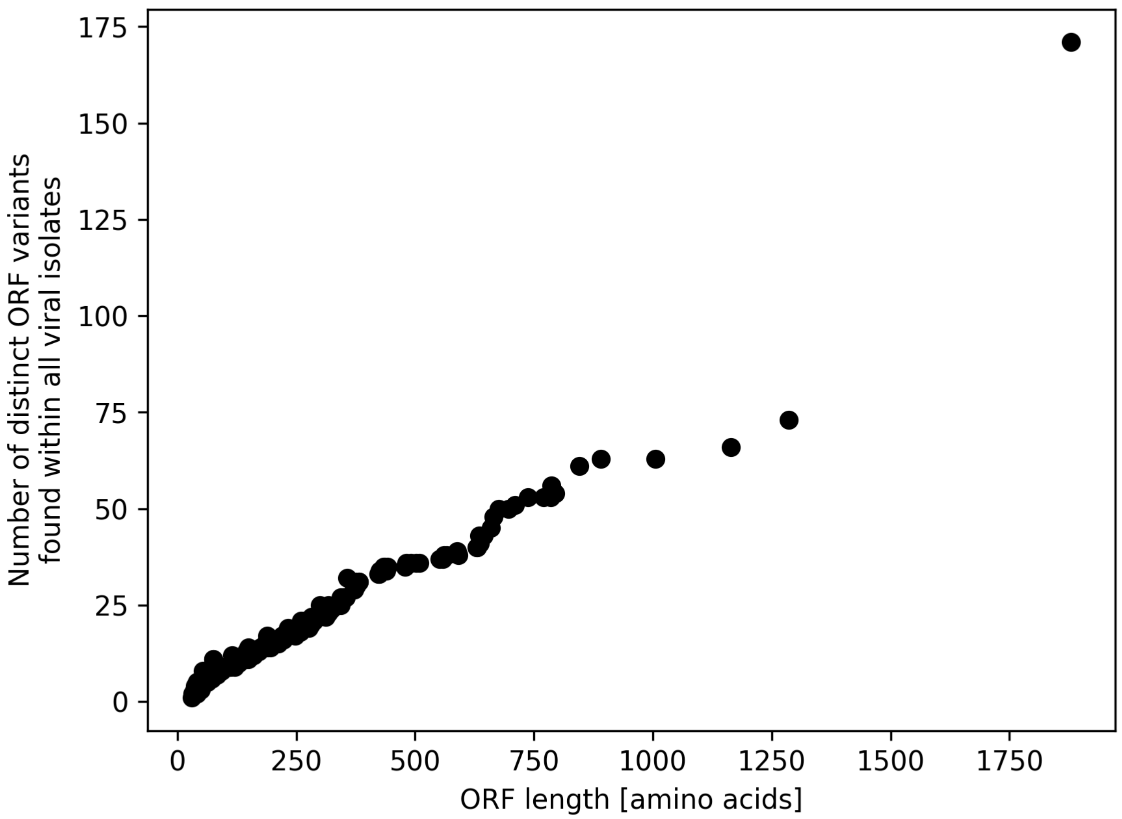

Figure S2 | Number of distinct ORF variants detected within 7,023 viral genome sequences. The number of different ORF variants (equal to the number of unique mutations or mutation-combinations) detected within all viral isolates is depicted on the y-axis, together with the lengths of the respective ORFs on the x-axis.

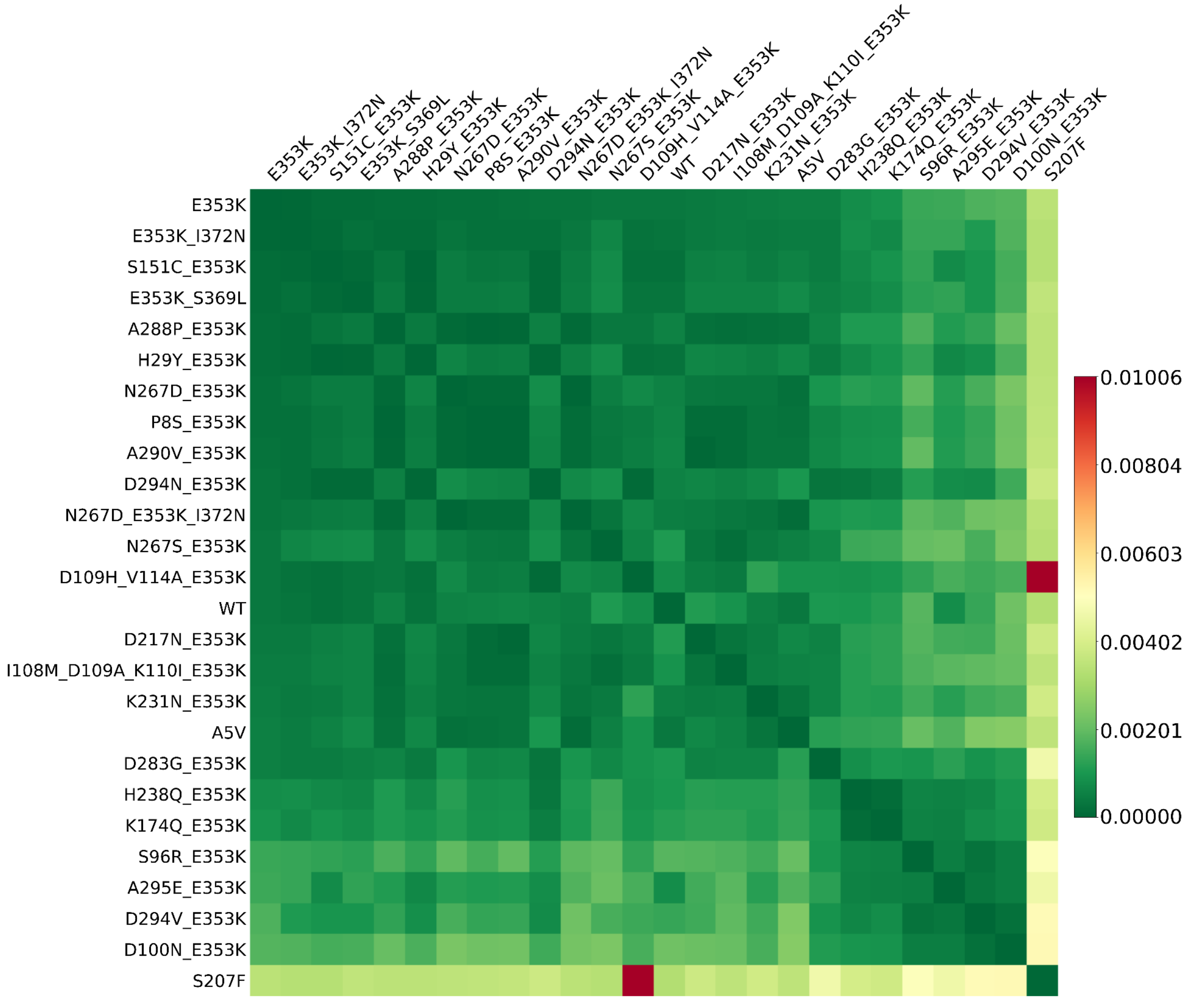

Figure S3 | Matching results of binding-site cavities calculated from homology models of all detected ID_6924 protein variants. The total matching score between Tecovirimat-binding-site cavities calculated from the homology models of all protein variants is depicted in this matrix. The total score is built from matching scores of cavity-properties such as electrostatics, aromaticity and point-cloud shape. The respective mutations compared to the NCBI reference protein sequence in each protein variant are labeled on the left-hand side and the top (multiple mutations in a single sequence are separated by "_") and "WT" refers to the NCBI reference protein sequence of phospholipase F13. The more similar two matched cavities are, the lower is the total score, and a score of 0 refers to identical cavities. Homology modeling, cavity-point-cloud creation and matchings were performed within the Catalophore™ Drug Solver platform.

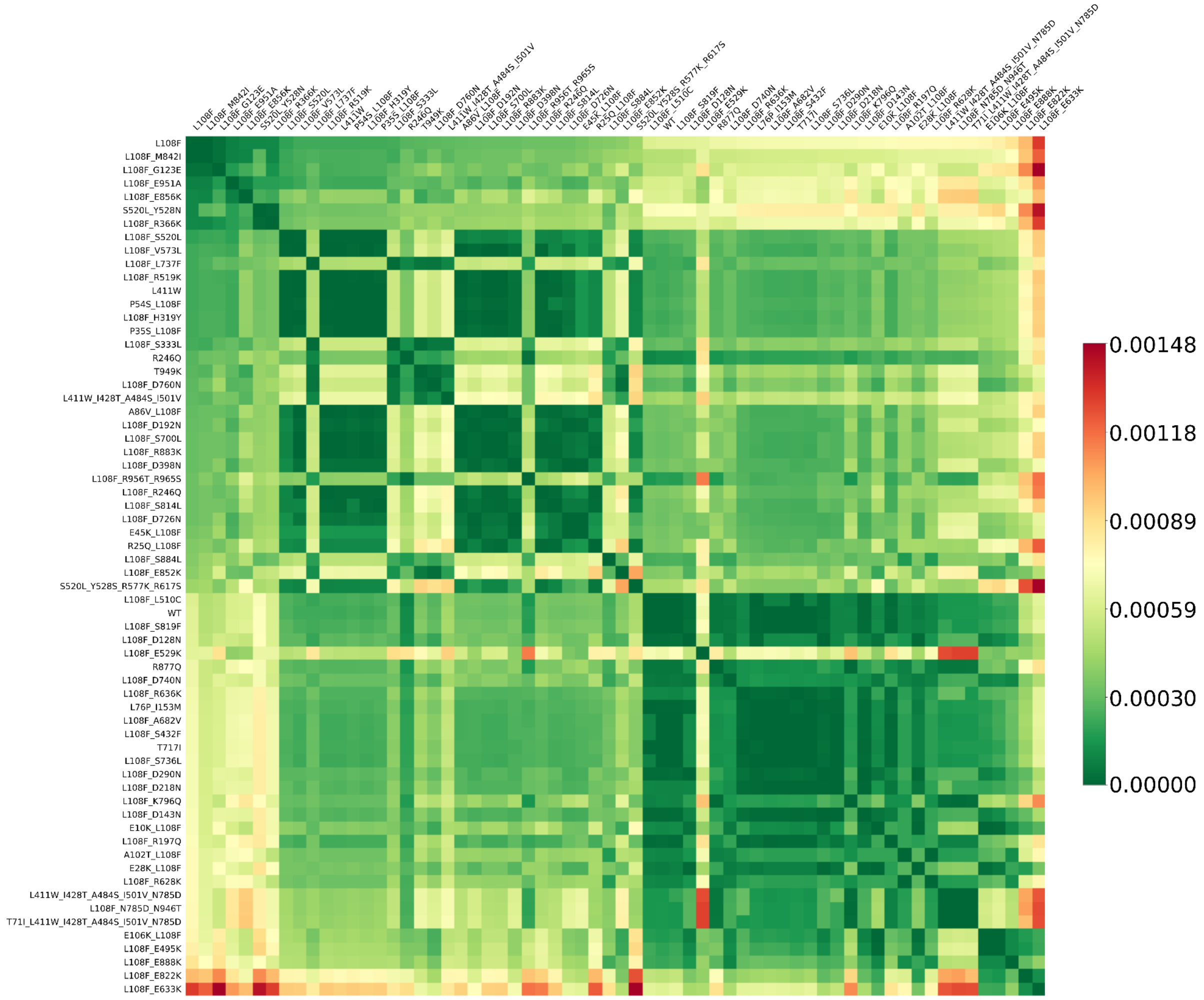

Figure S4 | Matching results of binding-site cavities calculated from homology models of all detected ID_8713 protein variants. The total matching score between Brincidofovir-binding-site cavities calculated from the homology models of all protein variants is depicted in this matrix. The total score is built from matching scores of cavity-properties such as electrostatics, aromaticity and point-cloud shape. The respective mutations compared to the NCBI reference protein sequence in each protein variant are labeled on the left-hand side and the top (multiple mutations in a single sequence are separated by "_") and "WT" refers to the NCBI reference protein sequence of DNA polymerase E9. The more similar two matched cavities are, the lower is the total score, and a score of 0 refers to identical cavities. Homology modeling, cavity-point-cloud creation and matchings were performed within the Catalophore™ Drug Solver platform.

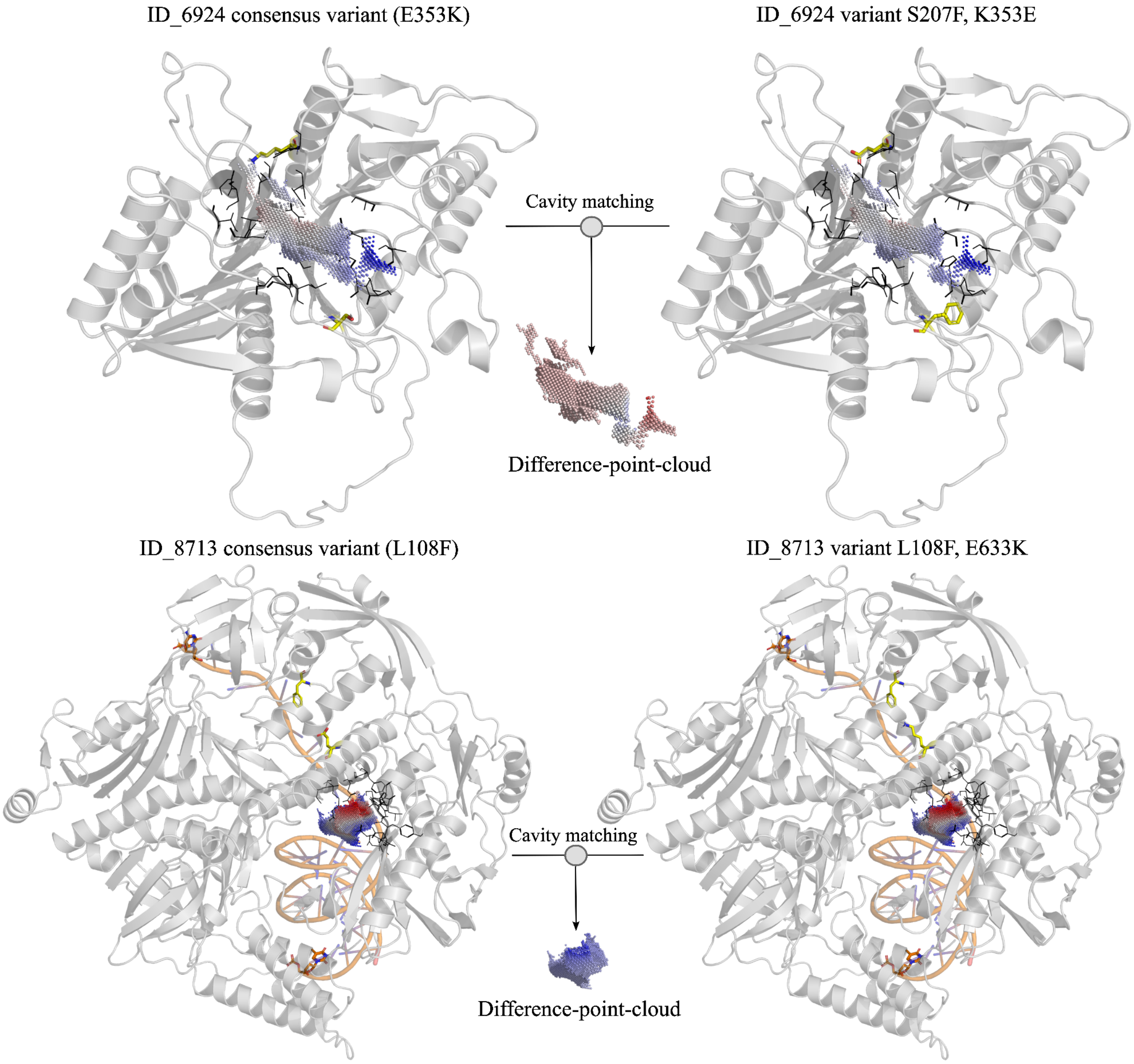

Figure S5 | Structural representation of the differences in electrostatics in the binding site of protein variants compared to the respective consensus ORF. The most different cavities compared to the cavity of the consensus protein variant were determined (Figure S3 and S4) and the respective difference-cavities are depicted here. Difference-point-clouds show the difference in electrostatics between the consensus variant and the variant on the right-hand side, colored from blue (-0.25) to white (0, no difference) to red (+0.25). The respective binding-site cavities used for the matching are colored by the electrostatics of their surroundings (blue-white-red spectrum ranging from -1 to +1). The respective mutated residues of each variant are shown as yellow sticks. Residues within 5 Å of the binding-site cavities are shown as black lines.

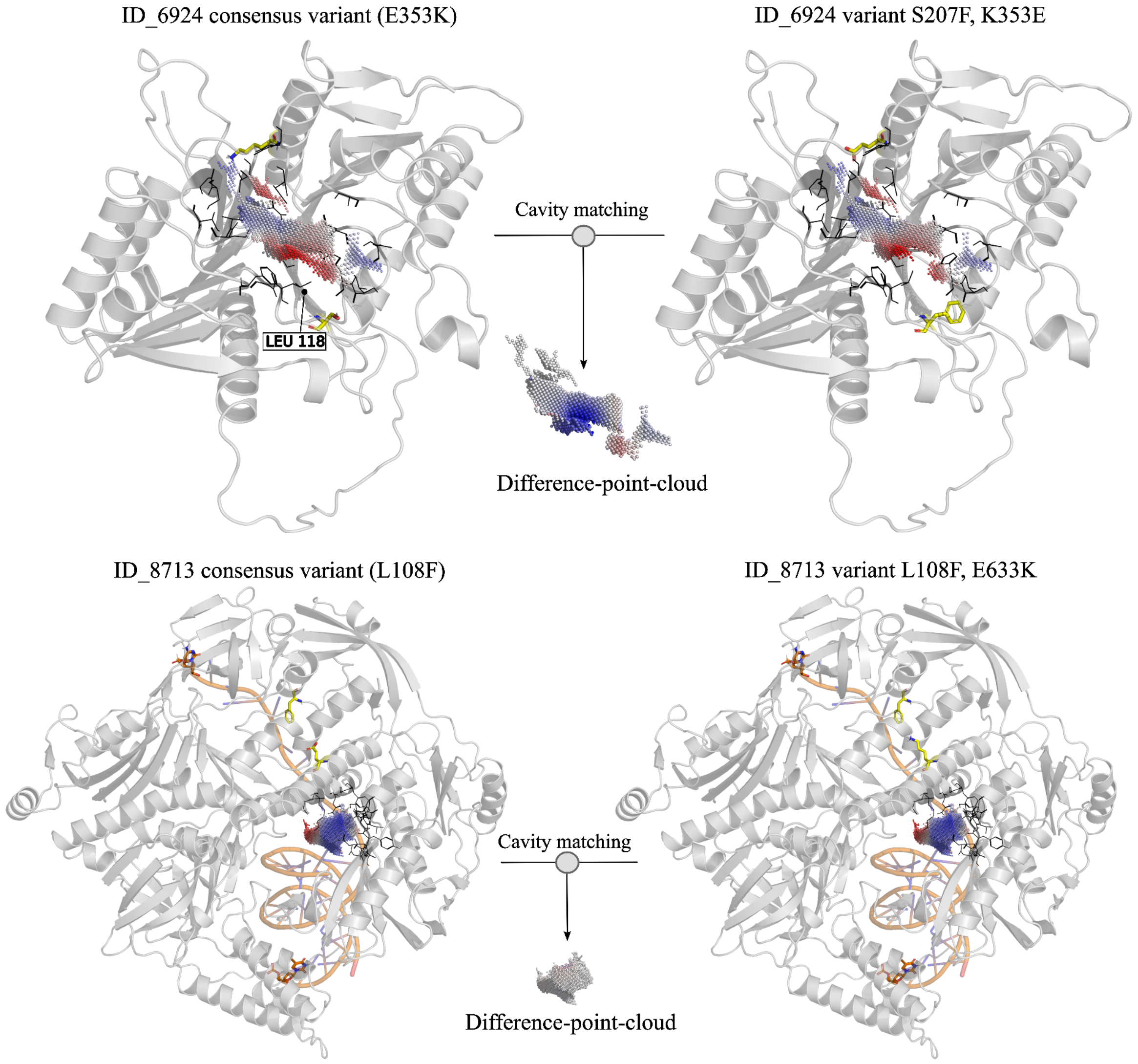

Figure S6 | Structural representation of the differences in hydrophobicity in the binding site of protein variants compared to the respective consensus ORF. The most different cavities compared to the cavity of the consensus protein variant were determined (Figure S3 and S4) and the respective difference-cavities are depicted here, showing the changes in hydrophobicity between the consensus variant and the variant on the right-hand side, colored from blue (-0.05) to white (0, no difference) to red (+0.05). The respective binding-site cavities used for the matching are colored by the hydrophobicity of their surroundings (blue-white-red spectrum ranging from -0.25 to +0.25). The respective mutated residues of each variant are shown as yellow sticks. Residues within 5 Å of the binding-site cavities are shown as black lines. Leucine at position 118 in ID_6924 is highlighted, as structural changes of this residue are responsible for the greatest difference in hydrophobicity.

SI References

1. Lin, Z. *et al.* Evolutionary-scale prediction of atomic level protein structure with a language model. 2022.07.20.500902 Preprint at https://doi.org/10.1101/2022.07.20.500902 (2022).
2. Gruber, K., Steinkellner, G. & Gruber, C. Determining novel enzymatic functionalities using three-dimensional point clouds representing physico chemical properties of protein cavities, WO2014080005A1. (2020).
3. Jumper, J. *et al.* Highly accurate protein structure prediction with AlphaFold. *Nature* **596**, 583–589 (2021).
